# Evaluation of a social determinants of health screening questionnaire and workflow pilot within an adult ambulatory clinic

**DOI:** 10.1186/s12875-021-01598-3

**Published:** 2021-12-24

**Authors:** Rachel L. Berkowitz, Linh Bui, Zijun Shen, Alice Pressman, Maria Moreno, Stephanie Brown, Anne Nilon, Chris Miller-Rosales, Kristen M. J. Azar

**Affiliations:** 1 Department of Public Health and Recreation, College of Health and Human Sciences, San José State University, One Washington Square, San José, CA 95192 USA; 2 Sutter Health Institute for Advancing Health Equity, 2121 N. California Blvd, Walnut Creek, CA 94596 USA; 3 Department of Nursing, School of Natural Sciences, Mathematics, and Engineering, California State University, Bakersfield, 9001 Stockdale Highway, Bakersfield, CA 93311 USA; 4 Sutter Health Center for Health Systems Research, 2121 N. California Blvd, Walnut Creek, CA 94596 USA; 5 Alta Bates Summit Medical Center, Sutter Health, 350 Hawthorne Ave., Oakland, CA 94609 USA; 6 Berkeley Emergency Medical Group, 2450 Ashby Ave., Berkeley, CA 94705 USA; 7 Sutter Health Population Health Services, 2121 N. California Blvd, Walnut Creek, CA 94596 USA; 8 Department of Health Care Policy, Harvard Medical School, 180 Longwood Avenue, Boston, MA 02115 USA; 9 Department of Epidemiology and Biostatistics, University of California San Francisco, 550 16th St., Second Floor, San Francisco, CA 94158 USA

**Keywords:** Social determinants of health screening, Ambulatory setting, Evaluation, RE-AIM, Implementation science, Intervention

## Abstract

**Background:**

There is increased recognition in clinical settings of the importance of documenting, understanding, and addressing patients’ social determinants of health (SDOH) to improve health and address health inequities. This study evaluated a pilot of a standardized SDOH screening questionnaire and workflow in an ambulatory clinic within a large integrated health network in Northern California.

**Methods:**

The pilot screened for SDOH needs using an 11-question Epic-compatible paper questionnaire assessing eight SDOH and health behavior domains: financial resource, transportation, stress, depression, intimate partner violence, social connections, physical activity, and alcohol consumption. Eligible patients for the pilot receiving a Medicare wellness, adult annual, or new patient visits during a five-week period (February-March, 2020), and a comparison group from the same time period in 2019 were identified. Sociodemographic data (age, sex, race/ethnicity, and payment type), visit type, length of visit, and responses to SDOH questions were extracted from electronic health records, and a staff experience survey was administered. The evaluation was guided by the Reach, Effectiveness, Adoption, Implementation, and Maintenance (RE-AIM) framework.

**Results:**

Two-hundred eighty-nine patients were eligible for SDOH screening. Responsiveness by domain ranged from 55 to 67%, except for depression. Half of patients had at least one identified social need, the most common being stress (33%), physical activity (22%), alcohol (12%), and social connections (6%). Physical activity needs were identified more in females (81% vs. 19% in males, *p* < .01) and at new patient/transfer visits (48% vs. 13% at Medicare wellness and 38% at adult wellness visits, *p* < .05). Average length of visit was 39.8 min, which was 1.7 min longer than that in 2019. Visit lengths were longer among patients 65+ (43.4 min) and patients having public insurance (43.6 min). Most staff agreed that collecting SDOH data was relevant and accepted the SDOH questionnaire and workflow but highlighted opportunities for improvement in training and connecting patients to resources.

**Conclusion:**

Use of evidence-based SDOH screening questions and associated workflow was effective in gathering patient SDOH information and identifying social needs in an ambulatory setting. Future studies should use qualitative data to understand patient and staff experiences with collecting SDOH information in healthcare settings.

**Supplementary Information:**

The online version contains supplementary material available at 10.1186/s12875-021-01598-3.

## Background

The social determinants of health (SDOH) are “the circumstances in which people are born, grow up, live, work and age, and the systems put in place to deal with illness...[which] are in turn shaped by a wider set of forces: economics, social policies, and politics” [1]. SDOH play a crucial role in shaping population health and health inequities [2, 3]. Differences in the SDOH experienced across racial/ethnic and socioeconomic groups contribute to inequities in health outcomes [2]. A large body of evidence demonstrates the influential role these factors play in health outcomes and the accessibility, availability, and experiences of healthcare [4].

There is increased recognition in clinical settings of the importance of documenting, understanding, and addressing patients’ SDOH in order to improve health and address persistent inequities [4–8]. In 2014, the National Academy of Medicine (NAM; formerly known as the Institute of Medicine) recommended the routine collection and use of patients’ social and behavioral determinants of health information in clinical settings across 12 social and behavioral domains – four that were already collected but not regularly used (race/ethnicity, tobacco use, alcohol use, residential address) and eight additional domains (educational attainment, financial resource strain, stress, depression, physical activity, social isolation, intimate partner violence, neighborhood median household income) [5, 9, 10]. A variety of SDOH screening tools have been developed [11] and some healthcare networks have implemented system-wide SDOH screening practices (e.g. Kaiser Permanente [12] and OCHIN Inc.’s community health center network [13]). However, the incorporation of SDOH screening into adult primary care is inconsistent across the country, and additional research is required to support effective implementation [14, 15].

A key dimension of such efforts is the study of the process, feasibility, and acceptability of developing and implementing a standard work for SDOH screening within clinical settings [10, 16–26]. Individual studies have described the process of developing SDOH tools [16–19, 25], implementing standard work flows [16–19], clinical staff perspectives on and acceptance of social needs screening approaches [20–24, 26], reach and completeness of patients’ recorded screening tool responses [10, 16, 22], patient time-to-completion for social risk screening questions [10], and positive identification of social needs among patients [19, 22, 23, 25]. There is a need to evaluate the process in its entirety, considering all of these elements in real-world clinical settings. However, few assessments have considered many or all of these elements simultaneously [13, 27, 28]. In addition, assessment of differences in the experience and use of SDOH screening across patient sociodemographic groups is not often incorporated into evaluations. To ensure that SDOH screening interventions can support addressing rather than perpetuating health inequities, such questions must be incorporated into evaluations.

As part of an initiative to address health inequities, Sutter Health, a large integrated health network in Northern California, piloted a screening questionnaire and workflow to gather patient-reported information on SDOH and health behaviors (hereafter referred to collectively as SDOH) and to refer patients with identified needs to social service support within and outside of the ambulatory healthcare setting. A team of medical care providers, clinic staff, and researchers utilized the evidence-based questions embedded within the Epic electronic health record (EHR) system [29] to ultimately develop an 11-question paper questionnaire and related workflow. We present an evaluation of the SDOH screening questionnaire and workflow and aim to understand its (1) reach in the eligible patient population and across sociodemographic groups, (2) impact on clinical care (i.e. visit length and the identification of SDOH needs across sociodemographic groups), and (3) staff perspectives in relation to the utility, appropriateness, barriers, and impact on patients of the pilot questionnaire and standard work.

## Methods

### Formative work

In November 2018, the Epic EHR module for SDOH was incorporated into Sutter Health’s system-wide Epic EHR platform. The module included 24 questions to identify SDOH needs across 10 domains (financial resource needs, transportation needs, stress, depression, intimate partner violence, social connections, physical activity, alcohol consumption, educational attainment, and marital status), based on the recommendations from NAM and the Centers for Medicare and Medicaid Services (CMS) [30]. The EHR module was adapted to a paper questionnaire (using the verbatim wording of Epic questions) and data collection procedure (hereafter referred to as “workflow”) through 4 week-long iterative plan-do-study-act (PDSA) cycles [31] in Fall 2019 at the pilot ambulatory clinic. The preliminary 24-question SDOH questionnaire and standard workflow were informally introduced and intermittently used within the pilot clinic and between January and mid-February 2020 to gain additional feedback for further refinement prior to the pilot. A focus group discussion with physicians and clinic staff (*N* = 9) in February 2020 finalized the questionnaire and standard workflow for the pilot.

### Description of the SDOH questionnaire and workflow

**Fig. 1:**
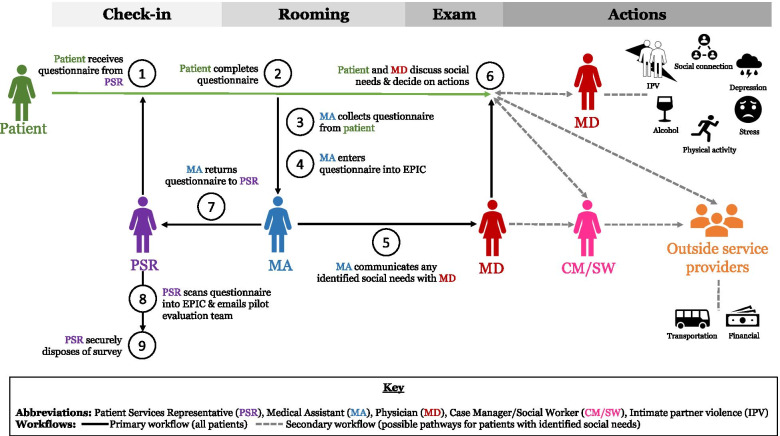
Workflow for Sutter Health SDOH Pilot. (1) Upon checking in, the patient receives the paper questionnaire from the PSR. (2) The patient completes the questionnaire in between checking in and being roomed by the MA. (3) The MA collects the questionnaire from the patient prior to the beginning of the MD visit. (4) The MA enters the paper questionnaire results into Epic. (5) The MA reviews which social needs have been identified based on the patient’s responses and discusses the identified social needs with the MD, which guides the MD in determining which potential actions to discuss with the patient during the exam. (6) The patient and the MD discuss social needs and actions and agree upon the next steps that will take place with the MD and/or a case manager/social worker. Beyond step 6, the secondary pilot workflow includes describes these next steps which would only occur for pilot patient participants with identified social needs who desire the actions. For six domains, the MD would work directly with the patient to support their ongoing needs. For two domains, the MD would refer the patient to a Sutter Health CM/SW. The CM/SW would in turn connect with the patient and identify relevant outside service providers to support the patient’s needs. (7) Once the paper questionnaire results have been entered and used, the MA returns the completed questionnaire to the PSR. (8) The PSR scans a copy of the paper questionnaire to Epic for data quality checks (and during the pilot specifically, emails that copy to the pilot evaluators). (9) The PSR securely disposes of the paper questionnaire

The SDOH questionnaire and workflow were piloted in a single ambulatory clinic from February 18th-March 25th, 2020 (pilot cut short due to COVID-19 pandemic). The questionnaire included 11 questions across eight SDOH domains: financial resource needs, transportation needs, stress, depression, intimate partner violence, social connections, physical activity, and alcohol consumption (Additional file 1). Physicians provided guidance on which responses for each question they felt identified a “need” and described the actions which identifying such a need would provoke from the following options: “No immediate action – document and monitor,” “Immediate physician intervention,” “Long-term physician management,” or “Referral to clinic case manager and/or social workers.” The final workflow describes the integrated data collection protocol from a patient checking into the clinic through the potential action steps following the identification of specific social needs (Fig. 1).

### Pilot evaluation

#### Evaluation conceptual framework

The pilot evaluation was guided by the dissemination and implementation science (D&I) Reach, Effectiveness, Adoption, Implementation, and Maintenance (RE-AIM) framework [32, 33]. The RE-AIM framework highlights key process and outcome evaluation metrics which are relevant for the development of an intervention in support of effective future implementation and scaling. We identified the most applicable constructs (Reach, Effectiveness, Adoption, and Implementation) within the RE-AIM framework to guide the pilot evaluation plan. Maintenance was not assessed due to the nature of this pilot study. Table 1 presents the RE-AIM constructs and corresponding evaluation questions, indicators, and data sources for the pilot evaluation.

**Table 1:** RE-AIM framework, evaluation objectives, indicators, and data sources

Applicable RE-AIM Constructs	Evaluation objectives	Indicator(s)	Data Source(s)
**Reach** “The absolute number, proportion, and representativeness of individuals who are willing to participate in a given...intervention...” p.3 [32]	Examine reach in the eligible patient population and across sociodemographic groups	Proportion of 2020 eligible patients who responded to any survey question	EHR data extraction
		Sociodemographic comparability of 2020 eligible patients based on survey completeness, overall and by domain	EHR data extraction
**Effectiveness** “The impact of an intervention on important outcomes, including potential negative effects...[as well as] heterogeneity of effects.” p.3 [32]	Understand effectiveness in identifying patient SDOH needs overall and across sociodemographic groups	Proportion of 2020 participating patients who had an identified SDOH need	EHR data extraction
		Sociodemographic comparability of 2020 participating patients based on identified SDOH need	EHR data extraction
	Discern staff perspectives on potential impact of pilot survey and standard work on patients	Proportion of staff who identify potential positive impacts on patients	Staff experience survey
		Proportion of staff who identify potential negative impacts on patients	Staff experience survey
	Discern physician perspectives on the impact of having social needs information on medical decision making	Proportion of physician staff respondents who agreed that having patients’ social needs information would influence medical decision making	Staff experience survey
**Adoption** “The absolute number, proportion, and representativeness of...intervention agents who are willing to initiate a program [and]...Reasons for adoption or non-adoption.” p.3 [32]	Discern staff perspectives in relation to the utility and appropriateness of the pilot survey and standard work	Proportion of staff who agree with the relevance of collecting social needs information	Staff experience survey
		Proportion of staff who agree with the utility of collecting social needs information	Staff experience survey
**Implementation** “At the setting level...the intervention agents’ fidelity to the various elements of an intervention’s protocol, including consistency of delivery as intended and the time required.” p.4 [32]	Assess impact on visit length across patient sociodemographic groups	Comparability of 2019 and 2020 visit lengths among eligible patients	EHR data extraction
		Sociodemographic comparability of visit length among 2020 eligible patients	EHR data extraction
	Discern staff perspectives on barriers to implementation of pilot survey and standard work	Proportion of staff who are knowledgeable about and confident in the implementation of the SDOH pilot survey and standard work	Staff experience survey
		Proportion of staff who experienced barriers to implementing the SDOH pilot survey and standard work	Staff experience survey

#### Pilot study setting and participants

The clinic conducts over 16,000 patient visits in Family Medicine and Internal Medicine annually. Eligible adult patients for screening were limited to those whose visits were classified as “Medicare Wellness”, “Health Maintenance Exam”, “New patient”, or “Transfer of care” in either the Internal Medicine or Family Medicine departments of the clinic. All patients with visits other than the four types of visits or whose visits were in different departments of the clinic were excluded from the pilot. Included visits are either one-time or annual wellness visits, and thus a patient would receive the questionnaire once within a 5-week pilot period.

#### Data sources

The pilot evaluation utilized two data sources. The first involved EHR data extraction. All eligible patients whose visits were between February 18th and March 25th in both 2019 (comparison group) and 2020 (pilot group) were identified. Visit lengths (i.e., time from check-in to rooming to beginning of exam to end of exam) and patient sociodemographic information (age, sex, race/ethnicity, and type of insurance used) were extracted for both groups. Though the SDOH questions were technically available in Epic during this time period in 2019, they were not consistently utilized. Responses to the SDOH questionnaire were therefore extracted from the visit records for the 2020 group only.

The second data source was a staff experience survey, made available through REDCap (Research Electronic Data Capture) [34, 35], a secure, web-based software platform hosted at Sutter Health, and emailed to all clinic staff. The survey questions were adapted from the survey developed by Schickendanz and colleagues [21] to assess clinical experiences with and attitudes towards SDOH screening in a large integrated health network. The pilot staff experience survey asked staff to reflect on the relevance and utility of collecting social needs information in the clinical setting, their knowledge of and confidence in implementing the standard work, what barriers they encountered, and the potential impacts of the pilot questionnaire and standard work on patients. Responses were collected between April 6th-20th 2020.

#### Evaluation metrics

Evaluation metrics corresponding to specific RE-AIM framework constructs and evaluation objectives are presented in Table 1. To assess the reach of the pilot, we described responsiveness to the SDOH questionnaire among the 2020 eligible patient group. We compared the distributions of sociodemographic groups between the 2019 and 2020 eligible patient groups to consider the representativeness of our pilot patient participants. To identify whether the reach varied by sociodemographic groups, we assessed whether there were differences between 2020 eligible patients who did and did not respond to the SDOH questionnaire across sociodemographic group and whether responsiveness for a particular domain varied by sociodemographic group.

Assessing the effectiveness of the pilot was restricted to only the 2020 eligible patient group. We described social needs identified across all domains and examined whether identified social needs (any and by specific domain) varied across sociodemographic groups. In addition, we describe staff survey responses to questions focused on the perceived positive or negative impacts of the pilot on patients and the perspectives of physicians as to whether or not having access to patients’ social needs information would influence their medical decision making.

To assess adoption, we focused on understanding the potential motivations of staff for adopting or not adopting the pilot questionnaire and standard work through responses to the staff experience survey. We describe staff responses to questions related to perceived relevance of the questionnaire and standard work in a clinical setting and perceived utility of the questionnaire in actually identifying unmet social needs and connecting patients with needed services.

To assess implementation, we examined visit length and staff perspectives on the pilot. To consider whether the pilot extended visit length, a chief concern among clinic staff, we assessed whether visit lengths – overall and for each component time period (i.e., checking-in, rooming, and exam) – differ between 2019 and 2020 eligible patients. We also assess whether visit lengths among 2020 eligible patients vary meaningfully across sociodemographic groups. Finally, we describe the degree to which staff felt knowledgeable of and prepared to implement the pilot, and the extent to which staff believed time, training, and/or resources were barriers to implementing the pilot, overall and by specific staff category.

#### Statistical analysis

Descriptive statistics (number and percent or mean and standard deviation) were conducted for all evaluation elements. Bivariate models (chi-square test, Fisher’s exact test, two-sample t-tests, or Kruskal-Wallis tests, as appropriate) were used to compare metrics across sociodemographic groups and years as applicable, with *p*-value < 0.05 considered statistically significant. Analysis was conducted in Stata [36] and R [37]. The pilot evaluation was determined to be a quality improvement project by the Sutter Health Institutional Review Board. No patient or staff protected health information (PHI) was accessed by Sutter Health researchers as a part of this quality improvement study, and no HIPPA waiver of consent was required.

## Results

There were 289 eligible patients during the pilot period (February 18th and March 25th, 2020), representing 16% of the total number of patients who visited the clinic during that time period. Twenty clinical staff members (87% of all staff) responded to the staff survey, including three physicians, seven MAs, five PSRs and five other staff (e.g., case managers).

### Reach

Table 2 compares the sociodemographic characteristics of 2019 and 2020 eligible patients. There were no statistically significant differences in sociodemographic characteristics (e.g., age, sex, race/ethnicity and payment type) between patients in the pilot and the comparison group in 2019. Fifty-seven percent (*N* = 164) of the 2020 eligible patients in the pilot were between 25 and 54 years of age, and 61% (*N* = 175) were female. Thirty-two percent of the 2020 patients (*N* = 92) self-identified as Hispanic, 31% (*N* = 89) as Non-Hispanic white, and 20% (*N* = 57) as Non-Hispanic Black. Nearly three-fourth of the 2020 patients (74%, *N* = 215) were privately insured, while 17% (*N* = 50) were Medicare patients.

**Table 2:** Characteristics of eligible patients, comparing February 18th-March 25th, 2019 and 2020

	2019	2020	***P-values***
	N(%)	N(%)	
**Eligible patients**	283 (100%)	289 (100%)	
**Age**			
18-24	30 (10.60)	35 (12.11)	0.9228^1^
25-34	68 (24.03)	63 (21.80)	
35-54	103 (36.40)	101 (34.95)	
55-64	37 (13.07)	40 (13.84)	
65+	45 (15.90)	50 (17.30)	
**Sex**			
Male	120 (42.40)	114 (39.45)	0.5261^1^
Female	163 (57.60)	175 (60.55)	
**Race/Ethnicity**			
Non-Hispanic white	92 (32.51)	89 (30.79)	–
Non-Hispanic Black	54 (19.08)	57 (19.72)	
Non-Hispanic Asian	10 (3.53)	25 (8.65)	
Hispanic/Latinx	103 (36.40)	92 (31.83)	
Non-Hispanic Native Hawaiian/ Pacific Islander	4 (1.41)	5 (1.73)	
Non-Hispanic American Indian/Alaska Native	1 (0.35)	0 (0.00)	
Multiple races	3 (1.06)	4 (1.38)	
Unknown/No Response	16 (5.65)	17 (5.88)	
**Insurance type**			
Medicaid	3 (1.06)	8 (2.77)	0.4167^1^
Medicare	49 (17.31)	50 (17.30)	
Private	221 (78.09)	215 (74.39)	
Self-Pay/Not Listed	4 (1.41)	8 (2.77)	
Other	6 (2.131.05)	8 (2.77)	
**Clinic**			
Family Medicine	171 (60.42)	184 (63.67)	0.4757^1^
Internal Medicine	112 (39.58)	105 (36.33)	
**Visit Type**			
New Patient/Transfer	192 (67.84)	188 (65.05)	0.7334^1^
Medicare Wellness	18 (6.36)	18 (6.23)	
Adult Wellness	73 (25.80)	83 (28.72)	

--- unable to calculate *p*-value

^1^*P*-values based on Chi-square test statistic

Table 3 describes patient responsiveness to the questionnaire by specific SDOH domain. The majority of eligible patients (83%) responded to at least one SDOH question. Responsiveness for seven of the eight domains, by domain, ranged from 55% (social connection) to 67% (alcohol). The questions regarding depression had the lowest response rate, with only 7 % of eligible participants completing at least one of the questions in this domain.

**Table 3:** Response to SDOH survey and identified SDOH needs

	***Any response to SDOH survey***^***a***^	***Identified SDOH need***^***b***^	***No response to SDOH survey***^***c***^
	N (%)	N (%)	N (%)
At least one domain	240 (83.04)	123 (51.25)	49 (16.96)
All domains	10 (3.46)	0 (0.00)	49 (16.96)
Financial resource need	152 (52.60)	11 (4.58)	137 (47.40)
Transportation	165 (57.09)	6 (2.50)	124 (42.91)
Alcohol	195 (67.47)	29 (12.08)	94 (32.53)
Physical activities	168 (58.13)	52 (21.67)	121 (41.87)
Stress	163 (56.40)	78 (32.50)	126 (43.60)
Depression	20 (6.92)	5 (2.08)	269 (93.08)
Social connections	159 (55.02)	15 (6.25)	130 (44.98)
Intimate partner violence	162 (56.06)	13 (5.42)	127 (43.94)

^a^Percentages based on denominator of all eligible patients of the pilot (*N* = 289)

^b^Percentages based on denominator of all patients who had any response to any questions on the SDOH survey entered into EHR (not including “No response”) (*N* = 240)

^c^No response to SDOH survey included “Declined” and “Blank”. Percentages based on denominator of all eligible patients of the pilot (*N* = 289)

We observed no statistically significant differences in responsiveness (response to at least one SDOH question) across sociodemographic groups and visit types (Additional file 2). The response rates for specific domains were uniform across domains and sociodemographic groups, with a few exceptions. Women were significantly more likely to respond to questions for the financial resource domain (54% vs. 46%; *p* < .05), the physical activity domain (66% vs. 34%, *p* < .05), and the intimate partner violence domain (66% vs. 34%, *p* < .05), compared to men. Though not statistically significant, women tended to provide more responses across the remaining domains. In addition, responsiveness was significantly different across age groups within the alcohol domain: 25- to 54-year-olds made up a significantly larger proportion of respondents (61%), and 65+ year-olds made up a significantly larger proportion of non-respondents (27%). Though significant differences were observed across age groups, insurance types, and visit types for responses to the depression domain, sample sizes were very small (only 20 people responded to the either of the domain questions).

### Effectiveness

Table 3 also describes whether a SDOH need was identified among all eligible patients who responded to at least one survey question (*N* = 240). The majority of participating patients (51%, *N* = 123) had at least one identified social need, and needs were identified within each domain. Among those who had any response to a given domain’s questions, the most commonly identified SDOH need was stress (33%, *N* = 78), followed by physical activity (22%, *N* = 52), alcohol (12%, *N* = 29), and social connections (6%, *N* = 15). Identification of at least one social need did not differ across sociodemographic groups and visit types (Additional file 2). Physical activity needs were identified for a significantly larger proportion of female patients compared to males (81% vs. 19%, *p* < .01) as well as at new patient/transfer visits compared to other visit types (48% at new patient/transfer vs. 13% at Medicare wellness and 38% at adult wellness visits, *p* < .05). Further, new/transfer patients made up a larger proportion of respondents without identified physical activity needs (68%) than other visit types. For three other domains, significant differences across sociodemographic groups were observed, but absolute numbers of patients with identified needs were small. A significant difference in reported intimate partner violence needs was observed by visit type: all reported intimate partner violence needs were among new/transfer patients (*N* = 13, 5.4%). All reported transportation needs (*N* = 6, 2.5%) were among Non-Hispanic Black individuals (*N* = 3) and Non-Hispanic Asian individuals (*N* = 3). All reported depression needs (*N* = 5, 2.1%) were among patients 55 and older, those with public insurance, and either Medicare Wellness or Adult Wellness visit types.

Table 4 presents the responses to the staff experience survey questions, across all responding staff and by staff category. With respect to the effectiveness of the pilot, 90% (*N* = 18) of respondents believed that social needs information could help improve patient care and health outcomes, and 90% (*N* = 18) felt that social needs information could improve therapeutic relationships with patients. Eighty percent (*N* = 16) of staff respondents reported being concerned that patients would feel uncomfortable answering the SDOH survey questions. Only one of the three responding physicians strongly agreed or agreed that having the social needs information would influence their medical decision making.

**Table 4:** Staff members’ perspectives by question

Statements	All (*N* = 20)	Physicians (*N* = 3)	MAs (*N* = 7)	PSRs (*N* = 5)	Other staff (*N* = 5)
	Strongly agree/Agree N (%)				
**Effectiveness**					
***Potential for SDOH survey and standard work to positively impact patients***					
Patients’ unmet social needs information could be used to improve patient care and health outcomes	18 (90%)	2 (67%)	6 (86%)	5 (100%)	5 (100%)
Patients’ unmet social needs information could be used to improve therapeutic relationship with patients	18 (90%)	2 (67%)	6 (86%)	5 (100%)	5 (100%)
Patients might feel uncomfortable answering questions about their unmet social needs	16 (80%)	2 (67%)	6 (86%)	4 (80%)	4 (80%)
Having access to patients’ unmet social needs information would influence physician’s medical decision	0 (0%)	1 (33%)	N/A	N/A	N/A
**Adoption**					
***Relevance of SDOH survey and standard work in clinical setting***					
Collecting social needs information is within the scope of clinical care	19 (95%)	3 (100%)	7 (100%)	5 (100%)	4 (80%)
Many patients in the clinic have unmet social needs that impact their health	16 (80%)	3 (100%)	4 (57%)	4 (80%)	5 (100%)
***Utility of SDOH survey and standard work for achieving intended aims***					
SDOH survey improves clinic’s ability to identify patients with unmet social needs	18 (90%)	2 (67%)	7 (100%)	5 (100%)	4 (80%)
The SDOH survey asks all relevant questions	16 (80%)	3 (100%)	5 (71%)	4 (80%)	4 (80%)
SDOH survey increases the likelihood that patients are connected with case management and social services	17 (85%)	2 (67%)	7 (100%)	4 (80%)	4 (80%)
**Implementation**					
***Knowledge and availability of needed resources in clinical setting to achieve positive impact for patients***					
I am aware of Sutter resources available to address patients’ social needs	10 (50%)	1 (33%)	3 (43%)	2 (40%)	4 (80%)
I am confident in my ability to help patients address their social needs	10 (50%)	0 (0%)	4 (57%)	2 (40%)	3 (60%)
I understand my role in offering the SDOH survey to patients	17 (85%)	2 (67%)	7 (100%)	4 (80%)	4 (80%)
***Barriers to implementing SDOH survey and standard work***	**Major barrier/Barrier (N(%))**				
Lack of time for patients to complete survey	15 (75%)	3 (100%)	6 (86%)	3 (60%)	3 (60%)
Lack of training about administering survey	4 (20%)	1 (33%)	1 (14%)	1 (20%)	1 (20%)
Lack of training about how to respond to social needs	9 (45%)	1 (33%)	3 (43%)	3 (60%)	2 (40%)
Lack of time to respond to social needs	13 (65%)	1 (33%)	5 (71%)	2 (40%)	3 (60%)
Lack of resources to address social needs	9 (45%)	2 (67%)	2 (29%)	2 (40%)	3 (60%)

### Adoption

As shown in Table 4, 95% (*N* = 19) of staff strongly agreed or agreed with the statement that collecting social needs information was within the scope of clinical care. Eighty percent (*N* = 16) of staff believed that the clinic patients would in fact have unmet social needs, including only 57% (*N* = 4) of MAs. These two questions capture a perception of the relevance of the SDOH survey and standard work.

Regarding the utility of the SDOH survey itself in the context of the standard work, 80% (*N* = 16) of staff felt the survey asked all relevant questions for assessing unmet social needs, 90% (*N* = 18) strongly agreed or agreed that the survey improved the clinic’s ability to identify patients with unmet social needs, and 85% (*N* = 17) believed that it would increase the likelihood of patients actually being connected to case managers or social services to address identified social needs.

### Implementation

As described in Table 4, 85% (*N* = 17) of staff understood their role in relation to the SDOH survey and standard work. Only 50% (*N* = 10) were aware of available Sutter Health resources to support, including one physician, three MAs, and two PSRs, the primary actors in the SDOH standard work. Similarly, only 50% (*N* = 10) of staff were confident in their ability to help patients address social needs, with no physicians reporting confidence.

All proposed potential barriers to implementing the standard work were identified as major barriers or barriers by multiple staff participants. Patients lacking time to complete the survey was identified as a barrier by the largest percent of staff (75%, *N* = 15), followed by staff lack of time to respond to social needs (65%, *N* = 13), staff lack of training about or resources for responding to social needs (both 45%, *N* = 9), and staff lack of training about administering the survey (20%, *N* = 4).

Table 5 compares the 2019 and 2020 eligible patient visit length. During the 2020 pilot, average length of visit across all eligible visits was 39.8 min, which was 1.7 min longer than during the same time period in 2019; however, the difference was not statistically significant. In looking at the specific segments of the visit, there was a small but statistically significant difference in the length of rooming (the time spent with the MA), with average 2020 rooming lasting approximately 0.7 min longer than average length of rooming in 2019 (*p* < .05). In comparison with the same time period in 2019, average length of visit in 2020 pilot was longer across all visit types from 1.2 to 3.7 min in difference, but the difference was not statistically significant. We did observe significantly longer rooming time among new/transfer patients by 1.36 min (*p* < .01) and significantly longer exam time among Medicare wellness patients by nearly 5 min (*p* < .05) in 2020 compared to 2019.

**Table 5:** Average lengths of visits (minutes), comparing 2019 and 2020

	2019	2020	***P*** values
	Mean (Standard Deviation)		
***All eligible visits (N = 572)***	***N = 283***	***N = 289***	
Whole visit	38.04 (14.77)	39.75 (13.88)	0.1537^1^
Check-in to rooming	7.59 (7.72)	7.57 (6.74)	0.1844^2^
Rooming (time with MA)	9.14 (6.85)	9.8 (5.16)	0.0409^2^*
Exam (time with provider)	17.09 (10.37)	16.22 (8.65)	0.6323^2^
***New patient or Transfer (N = 380)***	***N = 192***	***N = 188***	
Whole visit	38.79 (15.04)	40.66 (13.92)	0.2075^1^
Check-in to rooming	7.50 (7.93)	7.78 (7.29)	0.7187^1^
Rooming (time with MA)	9.21 (4.86)	10.57 (5.05)	0.0080^1^*
Exam (time with provider)	18.48 (10.3)	16.71 (8.62)	0.1819^2^
***Medicare Wellness (N = 36)***	***N = 18***	***N = 18***	
Whole visit	39.56 (15.36)	43.22 (14.21)	0.3751^3^
Check-in to rooming	9.5 (7.9)	7.94 (6.58)	0.5893^3^
Rooming (time with MA)	11.46 (4.05)	12.7 (6.8)	0.9495^3^
Exam (time with provider)	11.22 (4.46)	15.95 (9.85)	0.0314^3^*
***Health Maintenance Exam (N = 156)***	***N = 73***	***N = 83***	
Whole visit	35.7 (13.83)	36.93 (13.44)	0.5747^1^
Check-in to rooming	7.36 (7.13)	7.02 (5.39)	0.6102^2^
Rooming (time with MA)	8.37 (10.74)	7.43 (4.12)	0.7238^2^
Exam (time with provider)	14.89 (10.81)	15.17 (8.47)	0.5893^2^

^1^*P* values from two-sample t-test

^2^*P* values from Mann-Whitney test

^3^*P*-values from Mann-Whitney test using normal approximation due to small sample size and ties

*P < .05

Among the 2020 eligible patients, significant differences in visit length were observed across several sociodemographic groups and by visit type (Additional file 3). The average visit length was significantly longer among patients who are 65+ (43.36 min) compared to other age groups (*p* < .05) and on public insurance (43.64 min) compared to other insurance types (*p* < .05). Time for checking-in and rooming were also significantly different by insurance type: patients who were self-pay or having other insurance types had the longest checking-in time (10.4 min, *p* < .05), and patients with public insurance had the longest rooming time (12.2 min, *p* < .01)). Rooming is also significantly different across visit types, with the longest time among Medicare wellness patients (12.70 min, *p* < .01). Physician exam times were only significantly different by age group, with the 55-64 group had the longest exam time of 19.0 min (*p* < .01).

## Discussion

This pilot was the first effort to develop and evaluate a standard workflow using questions embedded within the Epic EHR system to gather patient SDOH information within the Sutter Health network. We found evidence of positive reach, effectiveness, adoption, and implementation while also identifying challenges which will require further in-depth investigation to support quality implementation across Sutter. Eighty-three percent of eligible patients responded to the questionnaire and responsiveness by SDOH domain ranged from 55 to 67%, except for depression. Fifty-one percent of the patients had at least one identified social need, the most common being stress (33%), physical activities (22%), alcohol (12%), and social connections (6%). Average length of visit during the pilot was 39.8 min, which was 1.7 min longer than that during the same time in previous year. Most staff agreed that collecting SDOH data was relevant and accepted the SDOH questionnaire and workflow but highlighted opportunities for improvement in training and connecting patients to resources. Though few, we did observe differences in reach, effectiveness, and implementation across patient sociodemographic groups. There is a need to better understand those observed differences and actively work to prevent inequitable implementation as this intervention is scaled. The findings for each specific RE-AIM dimension assessed will be discussed in greater depth below.

### Reach

We found no observable differences in sociodemographic characteristics between the participating patients in this pilot and the reference group of patients in 2019, indicating that the eligible patients in this pilot are likely similar to the patient population historically seen in the clinic. While 83% of the patients answered questions within at least one SDOH domain, only 3.5% of the patients completed all domains. Reasons for non-responsiveness may include discomfort with answering sensitive questions or the skipping of questions that patients did not feel were relevant to their lives. Much of current literature shows that patients perceive social risk screening as appropriate; however, research also emphasizes different factors which influence acceptability, such as trust in clinicians, clinical settings, and patients’ concern on privacy of social health data within their EHR [20, 38–41]. For example, a study by Cunningham & Sobell showed that adults feel that it is appropriate to be asked questions on alcohol use but may feel uncomfortable or underreport [42]. Stigma or shame was cited as barriers to disclosure of social needs like food insecurity [43]. The overall non-responsiveness to the depression domain questions (with only 7% of eligible patients responding) may also be indicative of stigma against mental illness, though evidence from other clinical sites suggests that depression screening is increasingly perceived as positive [44]. Further investigation is needed to understand why depression screening in the context of the overall SDOH questionnaire was unsuccessful. Additionally, our finding that females were more likely than males to respond to survey questions may be indicative of females’ greater recognition of the importance of screening. A study of patient perspectives on SDOH screening by Rogers and colleagues found that females were significantly more likely than males to agree that social needs screenings were necessary and should be a part of healthcare settings [45]. Overall, these findings emphasize the importance of patient-centered implementation of social risk screening. Due to the scope of this evaluation, we were not able to understand patients’ perspectives regarding answering the SDOH questionnaire. Future iterations of this intervention should incorporate a qualitative component to understand patients’ experiences of and feelings about answering the SDOH questions in the context of this standard workflow [45, 46].

### Effectiveness

Half of the participating patients had at least one identified social need, with the most commonly identified social needs being stress, physical activities, alcohol, and social connections. These findings are similar to those reported in other studies that used different SDOH screening tools and workflows. A study by Page-Reeves and colleagues of patients in family medicine clinics also found identified 46% of patients having social needs [19]. A recent study by Tong and colleagues on a target population with a higher risk of having social needs reported a higher proportion (71–86%) of patients who screened positive for social needs, with the most common social needs included physical activities, dental, and alcohol use [23]. Despite the SDOH questions being available at Sutter Health in Epic since 2019, these questions were not used by physicians before the pilot. The fact that SDOH needs were identified in this pilot indicates the importance of having a standard workflow to gather SDOH information in a systematic way [15, 18]. Although we found certain differences in identified social needs across sociodemographic characteristics, small sample sizes limit the interpretability of these findings. Given the dearth of literature on whether identified social needs from screenings in clinical settings differ across patient populations, future research with a larger sample size and an incorporated qualitative component would provide the opportunity of examining potential disparities in the identification of social needs.

Overall, there was a strong support from staff in using the SDOH questionnaire and workflow to collect SDOH information as they were perceived to be important and effective in identifying social needs among patients. Although evidence was mixed, recent studies also show that social risk screening is perceived to be important by clinicians and healthcare team [23, 47, 48]. In this pilot, staff identified one possible challenge to effectively being able to collect SDOH information: that patients could feel uncomfortable answering the questions. Previous studies also reported concerns from clinicians related to ensuring that screening was done empathetically, without negative judgement, and with attention to privacy protections [19]. A qualitative study by Byhoff and colleagues found that patients actually felt more “cared for” or “listened to” when asked about social needs within a clinical setting, while emphasizing the need for “empathy” and “compassion” from staff conducting the screenings [46]. Accordingly, future implementations of this intervention must prioritize understanding patients’ experiences with answering the SDOH questions.

In the pilot’s standard workflow, physicians are a key gatekeeper of effectiveness, as they directly impact the translation of identified needs to action (whether within the exam space or through referral to case managers or social workers). Despite feeling that social needs information could help improve therapeutic relationship with patients, only one of the three responding physicians reported that having the information would influence their medical decision-making. Other studies found that knowing patients’ social needs not only improved patient-provider communications but also changed what clinicians do [23], such as providing more exercise and dietary counseling, being mindful of medication costs when prescribing, and helping with transportation to access to clinics. As only three physicians completed the staff survey, it was challenging to interpret physicians’ perceptions on changes of medical decisions without in-depth conversation, suggesting the utility of qualitative research with physicians in future pilots.

### Adoption

The overwhelming majority of surveyed staff indicated that SDOH screening was relevant for their specific clinical population and within the scope of clinical care more generally. These findings align with the overarching recognition in health care delivery that understanding and engaging with patients’ SDOH needs should be incorporated into primary care settings [5, 15]. The majority of staff also felt the survey itself asked relevant questions and would be useful to support patients in connecting with resources to address social needs. In their study of 258 clinicians (physicians, social workers, nurses, and pharmacists) at Kaiser Permanente Southern California, Schickendanz et al., similarly found that the majority of those surveyed agreed that social need screening should be incorporated into clinical care and that knowing such information could be beneficial to patients [21]. These findings from our pilot site suggest the staff’s willingness to initiate SDOH screening. They also suggest that implementing plan-to-study-act (PDSA) cycles with a small group of staff before launching the pilot could be a potential strategy to engage staff in the pilot and their willingness to initiate SDOH screening. As the SDOH questionnaire and workflow is adapted and implemented at other Sutter Health sites, attention should be paid to any differences in adoption across locations so that factors which best facilitate adoption in the Sutter Health network can be identified.

### Implementation

Because our study assesses a pilot program, we cannot speak to fidelity to the intervention with respect to the implementation of the SDOH questionnaire and workflow in other sites. Rather, our results speak to potential barriers that could hinder implementation at future sites – confusion regarding components of the standard workflow and the impact of the workflow on time [21, 22, 26]. While most staff felt they understood their role in the standard work, staff were less knowledgeable about and lacked training on available system resources to support patients and were also less confident in the ability to act on identified needs. Other studies have identified clinicians’ concerns that SDOH screening may not ultimately be helpful due to lack of availability or knowledge of, or access to, resources to address patients’ social needs as a potential barrier to implementation [23, 49]. The uncertainty expressed by staff speaks to the importance of incorporating information on this dimension of the workflow into staff trainings, including for staff who will not be directly supporting patients with next steps.

Time – specifically the additional time needed by patients and staff to complete the SDOH workflow – was a key concern for staff in the pilot. These concerns have also been identified in other studies of clinician perspectives [21, 26]. Our study did find that the average length of visits among eligible patients in 2020 was slightly longer than those in 2019, but the difference was not statistically significant. The longest added time period was the statistically significant difference of 5 min in exam time for Medicare wellness patients. One possible explanation for the exam-time difference for Medicare wellness patients is that Medicare wellness patients may have greater social needs, and so the results of the SDOH questionnaire may ultimately require more time with the physician to identify next steps. However, we did not observe that Medicare wellness patients had significantly more needs identified as compared with patients of other visit types. Medicare wellness patients may also have more complex health needs, and the evaluation for these patients may take more time due to Medicare requirements. Another possible explanation is the potential for particularly long visits to have a greater effect when comparing average values for a small number of visits (6% of our 2020 pilot population). Medicare wellness visits did have the most variability in lengths of the whole visit, rooming, and exam. Similarly, we observed small but significant differences in average visit lengths and lengths of specific sections of the visit across different sociodemographic groups (by age, insurance type, and visit type). However, without in-depth conversations with patients and providers, we cannot understand the impact these average length increases had on the experience of care, nor can we determine what may have been gained or lost by incorporated the SDOH workflow into the limited available time. In a qualitative study of social risk screening among patients and caregivers, participants expressed concern for the addition of the screening to already overworked clinician’s schedules [46]. In future iterations of the intervention, close attention should be paid to the quantitative and qualitative impact of the SDOH workflow on time so that adjustments may be made, whether to visit lengths or to the SDOH workflow, to minimize the time-pressure felt by clinic staff and patients and maximize the impact of the intervention.

### Equity considerations

This pilot evaluation adds to the limited literature of SDOH assessments in clinical settings that examine whether there are disparities in screening and identification of social needs across different patient populations. Though observed in a small sample size, we identified some differences in reach, effectiveness, and implementation across patient sociodemographic groups. Understanding potential reasons for those differences could improve equitable implementation of SDOH assessment among patient groups. As addressing social needs is a strategy for reducing health inequities, SDOH assessment should be disseminated in clinical settings with a mindful approach that minimizes the potential disparities across patient sociodemographic groups.

### Strengths and limitations

As the first study in the Sutter Health network to assess the incorporation of an SDOH questionnaire and workflow into a primary care setting, this evaluation not only provides feedback for further development of the intervention within the pilot clinic but also lays the groundwork for system-wide scale-up. Rather than assessing a single dimension of the intervention, this study synthesizes data from different sources to evaluate multiple elements of the intervention simultaneously. Through the use of the RE-AIM framework, this study presents a systematic approach to assessing social risk screening interventions that can be replicated by other clinics. Our study’s focus on identifying observable differences across sociodemographic groups also sets an important precedent for future studies to center considerations of equity throughout evaluations of SDOH screening interventions.

There are also important limitations of this pilot evaluation. Due to insufficient resources, we were unable to assess the effectiveness of the standard workflow in addressing identified patient social needs, a gap in the evaluation which must be prioritized in future intervention studies. A growing body of literature is examining the impact of screening for social risks in clinical settings on patient access to resources, healthcare experiences, and health, with evidence suggesting that such screenings are beneficial for patients [19, 49–51]. As we could not examine how patient’s social needs were addressed in this pilot, future pilots should prioritize assessment of referrals, receipt of social services, and overall impact on patients [28]. In addition, our study timeline was impacted by the onset of the COVID-19 pandemic, which in turn limited our sample size with respect to comparing metrics across sociodemographic groups. Relatedly, as the pilot only focused on a single clinic, small sample sizes precluded inferential statistics across staff groups. Also, as previously discussed, future studies should examine the reasons for non-responsiveness to SDOH questionnaires in order to suggest effective ways for collecting SDOH information. Finally, due to the COVID-19 pandemic, we were unable to complete the intended patient experience component of the evaluation. Gaining a deeper understanding of patient perspectives remains a priority for future implementation.

## Conclusions

As healthcare continues to prioritize understanding and addressing patients’ social needs within clinical settings, pragmatic research considering the processes, impacts, and challenges of implementing SDOH screening workflows is vital [14, 15]. This assessment of a pilot within a Sutter Health primary care clinic provides an in-depth examination of an SDOH questionnaire and standard workflow intervention that can benefit dissemination within and outside of the Sutter Health network. Through effective identification of and support for addressing social needs, primary care settings can better provide holistic, comprehensive, and effective care to all patients.

## Supplementary Information

**Additional file 1.** Threshold and Actions for 11-Question SDOH Pilot Questionnaire.

**Additional file 2.** Response to SDOH survey and SDOH identified need across 2020 eligible patient sociodemographic characteristics, by any domain and by specific domain.

**Additional file 3.** Length of visit by 2020 eligible patient sociodemographic characteristics.

## Data Availability

The datasets generated and analysed during the current study are not publicly available because they necessarily contain PHI and were generated for the quality improvement project so long as the data remained on the Sutter Health secure network. A limited version of the data sets may be available from the corresponding author on reasonable request.

## Abbreviations

SDOH: Social determinants of health
RE-AIM: Reach, Effectiveness, Adoption, Implementation, Maintenance
EHR: Electronic health record
PHI: Protected health information
NAM: National Academy of Medicine
PSR: Patient Services Representative
MA: Medical assistant
MD: Doctor of medicine
CM/SW: Case manager/Social worker
D&I: Dissemination and Implementation Science
