# Supplementary material for: Evaluation of a social determinants of health screening questionnaire and workflow pilot within an adult ambulatory clinic
Source: BMC Fam Pract. 2021 Dec 24;22:256. doi: 10.1186/s12875-021-01598-3 (PMC8708511; doi:10.1186/s12875-021-01598-3)
Supplement: Supplementary file 1 — Additional file 1. Threshold and Actions for 11-Question SDOH Pilot Questionnaire. [file 12875_2021_1598_MOESM1_ESM.docx]

**Additional File 1: Threshold and Actions for 11-Question SDOH Pilot Questionnaire**

| **Domain** | **Question (in order on questionnaire)** | **Threshold for Action** | **Action(s)** |
| --- | --- | --- | --- |
| **Alcohol consumption** | 1. **How often do you have a drink containing alcohol?**  - Never [1] - Monthly or less [2] - 2-4 times a month [3] - 2-3 times a week [4] - 4 or more times a week [5] - Declined to Answer [98] | - 2-3x/week [4] - 4 or more times a week [5] | - Immediate physician intervention [5] - Long-term physician management [4,5] |
| **Financial resource needs** | 1. **How hard is it for you to pay for the very basics like food, housing, medical care, and heating?**  - Very hard [1] - Hard [2] - Somewhat hard [3] - Not very hard [4] - Not hard at all [5] - Declined to Answer [98] - Don’t know [99] | - Very hard [1] - Hard [2] - Somewhat hard [3] | - Referral to case manager/social worker |
| **Transportation needs** | 1. **Has the lack of transportation kept you from medical appointments or from getting medications?**  - Yes [1] - No [2] - Declined to Answer [98] - Don’t know [99] | - Yes [1] | - Referral to case manager/social worker |
| **Physical activity** | 1. **On average, how many days per week do you engage in moderate to strenuous exercise (like walking fast, running, jogging, dancing, swimming, biking, or other activities that cause a light or heavy sweat)?**  - 0 days [0] - 1 day [1] - 2 days [2] - 3 days [3] - 4 days [4] - 5 days [5] - 6 days [6] - 7 days [7] - Declined to Answer [98] - Don’t know [99] | - 0 days [0] | - Long-term physician management |
| **Stress** | 1. **Do you feel stress - tense, restless, nervous, or anxious, or unable to sleep at night because your mind is troubled all the time - these days?**  - Not at all [1] - Only a Little [2] - To Some Extent [3] - Rather Much [4] - Very Much[5] - Declined to Answer [98] | - To Some Extent [3] - Rather Much [4] - Very Much [5] | - Immediate physician intervention - Long-term physician management |
| **Social connections** | 1. **In a typical week, how many times do you talk on the telephone with family, friends, or neighbors?**  - Never [1] - Once a week [2] - Twice a week [3] - Three times a week [4] - More than three times a week [5] - Declined to Answer [98] | - Never [1] | - Document and monitor- no immediate action |
| **Social connections** | 1. **How often do you get together with friends or relatives?**  - Never [1] - Once a week [2] - Twice a week [3] - Three times a week [4] - More than three times a week [5] - Decline to Answer [98] | - Never [1] | - Document and monitor- no immediate action |
| **Intimate partner violence** | 1. **Within the last year, have you been humiliated or emotionally abused in other ways by your partner or ex-partner?**  - Yes [1] - No [2] - Declined to Answer [98] | - Yes [1] | - Immediate physician intervention - Long-term physician management - Referral to case manager/social worker |
| **Intimate partner violence** | 1. **Within the last year, have you been kicked, hit, slapped, or otherwise physically hurt by your partner or ex-partner?**  - Yes [1] - No [2] - Decline to Answer [98] | - Yes [1] | - Immediate physician intervention - Long-term physician management - Referral to case manager/ social worker |
| **Depression** | 1. **Within the last two weeks, have you had little interest or pleasure in doing things?**  - Yes [1] - No [0] - Decline to Answer [LEAVE BLANK] | - Yes [1] | - Immediate physician intervention - Long-term physician management |
| **Depression** | 1. **Within the last two weeks, have you been feeling down, depressed, or hopeless?**  - Yes [1] - No [0] - Decline to Answer [LEAVE BLANK] | - Yes [1] | - Immediate physician intervention - Long-term physician management |
