# Supplementary material for: Evaluation of a social determinants of health screening questionnaire and workflow pilot within an adult ambulatory clinic
Source: BMC Fam Pract. 2021 Dec 24;22:256. doi: 10.1186/s12875-021-01598-3 (PMC8708511; doi:10.1186/s12875-021-01598-3)
Supplement: Supplementary file 2 — Additional file 2. Response to SDOH survey and SDOH identified need across 2020 eligible patient sociodemographic characteristics, by any domain and by specific domain. [file 12875_2021_1598_MOESM2_ESM.docx]

**Additional File 2: Response to SDOH survey and SDOH identified need across 2020 eligible patient sociodemographic characteristics, by any domain and by specific domain.**

**a. Response to SDOH survey and SDOH identified need across 2020 eligible patient sociodemographic characteristics: Any domain**

|  | **Any domain responses^1^** | | | **Any domain identified need^2^** | | | |  |
| --- | --- | --- | --- | --- | --- | --- | --- | --- |
|  | **Responded** | **No response^3^** | ***p* values^4^** | **Yes** | **No** | ***p* values^4^** | |  |
|  | N (%) | N(%) |  | N(%) | N(%) |  |  |  |
| **All eligible patients** | 240 (83.0) | 49 (17.0) |  | 123 (51.3) | 117 (48.8) |  | |  |
| **Age** |  |  |  |  |  |  | |  |
| 18 – 24 | 30 (12.5) | 5 (10.2) | 0.79 | 17 (13.8) | 13 (11.1) | 0.40 | |  |
| 25 – 34 | 53 (22.1) | 10 (20.4) |  | 22 (17.9) | 31 (26.5) |  |  |  |
| 35 – 54 | 86 (35.8) | 15 (30.6) |  | 44 (35.8) | 42 (35.9) |  |  |  |
| 55 – 64 | 32 (13.3) | 8 (16.3) |  | 16 (13.0) | 16 (13.7) |  |  |  |
| 65+ | 39 (16.3) | 11 (22.5) |  | 24 (19.5) | 15 (12.8) |  |  |  |
| **Gender** |  |  |  |  |  |  | |  |
| Male | 89 (37.1) | 25 (51.0) | 0.10 | 44 (35.8) | 45 (38.5) | 0.77 | |  |
| Female | 151 (62.9) | 24 (49.0) |  | 79 (64.2) | 72 (61.5) |  |  |  |
| **Race/Ethnicity** |  |  |  |  |  |  | |  |
| Non-Hispanic white | 72 (30.0) | 17 (34.7) | 0.13 | 44 (35.8) | 28 (23.9) | 0.11 | |  |
| Non-Hispanic Black | 51 (21.3) | 6 (12.2) |  | 27 (22.0) | 24 (20.5) |  |  |  |
| Non-Hispanic Asian | 20 (8.3) | 5 (10.2) |  | 8 (6.5) | 12 (10.3) |  |  |  |
| Hispanic/Latinx | 72 (30.0) | 20 (40.8) |  | 36 (29.3) | 36 (30.8) |  |  |  |
| Other^5^ | 25 (10.4) | 1 (2.0) |  | 8 (6.5) | 17 (14.5) |  |  |  |
| **Insurance type** |  |  |  |  |  |  | |  |
| Public^6^ | 46 (19.2) | 12 (24.5) | 0.66 | 28 (22.8) | 18 (15.4) | 0.26 | |  |
| Private | 181 (75.4) | 34 (69.4) |  | 90 (73.2) | 91 (77.8) |  |  |  |
| Self-pay/Not Listed/Other | 13 (5.4) | 3 (6.1) |  | 5 (4.1) | 8 (6.8) |  |  |  |
| **Visit type** |  |  |  |  |  |  | |  |
| New Patient/Transfer | 153 (63.8) | 35 (71.4) | 0.41 | 76 (61.8) | 77 (65.8) | 0.74 | |  |
| Medicare Wellness | 17 (7.1) | 1 (2.0) |  | 10 (8.1) | 7 (6.0) |  |  |  |
| Adult Wellness | 70 (29.2) | 13 (26.5) |  | 37 (30.1) | 33 (28.2) |  |  |  |
| 1. Percentages based on denominator of all eligible patients of the pilot (N = 289) or column total  2. Percentages based on denominator of all patients who had any response to any questions on the SDOH survey entered into EHR (not including “No response") (N=240) or column total  3. No response to SDOH survey included “Declined” and “Blank”  4. p-values based on Chi-square test statistic or Fisher’s exact test  5. “Other” race/ethnicity category includes American Indian/Alaska Native individuals, Native Hawaiian/Pacific Islander individuals, individuals with multiple racial identities, and individuals whose racial/ethnic identity is unknown or not reported. These groups were combined due to small numbers.  6. “Public” insurance includes both Medicare and Medicaid users  *p<0.05 | | | | | | |  | |

**b. Response to SDOH survey and SDOH identified need across 2020 eligible patient sociodemographic characteristics: Financial resource domain**

|  | **Financial domain responses^1^** | | | **Financial domain identified need^2^** | | | |  |
| --- | --- | --- | --- | --- | --- | --- | --- | --- |
|  | **Responded** | **No response^3^** | ***p* values^4^** | **Yes** | **No** | ***p* values^4^** | |  |
|  | N (%) | N(%) |  | N(%) | N(%) |  |  |  |
| **All eligible patients** | 152 (52.6) | 137 (47.4) |  | 11 (4.6) | 229 (95.4) |  | |  |
| **Age** |  |  |  |  |  |  | |  |
| 18 – 24 | 22 (14.5) | 13 (9.5) | 0.67 | 1 (9.1) | 29 (12.7) | 0.72 | |  |
| 25 – 34 | 34 (22.4) | 29 (21.2) |  | 1 (9.1) | 52 (22.7) |  |  |  |
| 35 – 54 | 53 (34.9) | 48 (35.0) |  | 4 (36.4) | 82 (35.8) |  |  |  |
| 55 – 64 | 19 (12.5) | 21 (15.3) |  | 2 (18.2) | 30 (13.1) |  |  |  |
| 65+ | 24 (15.8) | 26 (19.0) |  | 3 (27.3) | 36 (15.8) |  |  |  |
| **Gender** |  |  |  |  |  |  | |  |
| Male | 51 (33.6) | 63 (46.0) | 0.04* | 4 (36.4) | 85 (37.1) | 1.00 | |  |
| Female | 101 (66.4) | 74 (54.0) |  | 7 (63.6) | 144 (62.9) |  |  |  |
| **Race/Ethnicity** |  |  |  |  |  |  | |  |
| Non-Hispanic white | 48 (31.6) | 41 (29.9) | 0.63 | 4 (36.4) | 68 (29.7) | 0.17 | |  |
| Non-Hispanic Black | 34 (22.4) | 23 (16.8) |  | 5 (45.5) | 46 (20.1) |  |  |  |
| Non-Hispanic Asian | 13 (8.6) | 12 (8.8) |  | 1 (9.1) | 19 (8.3) |  |  |  |
| Hispanic/Latinx | 46 (30.3) | 46 (33.6) |  | 1 (9.1) | 71 (31.0) |  |  |  |
| Other^5^ | 11 (7.2) | 15 (10.9) |  | 0 (0.00) | 25 (10.9) |  |  |  |
| **Insurance type** |  |  |  |  |  |  | |  |
| Public^6^ | 31 (20.4) | 27 (19.7) | 0.77 | 5 (45.5) | 41 (17.9) | 0.09 | |  |
| Private | 114 (75.0) | 101 (73.7) |  | 6 (54.5) | 175 (76.4) |  |  |  |
| Self-pay/Not Listed/Other | 7 (4.6) | 9 (6.6) |  | 0 (0.00) | 13 (5.7) |  |  |  |
| **Visit type** |  |  |  |  |  |  | |  |
| New Patient/Transfer | 93 (61.2) | 95 (69.3) |  | 2 (72.7) | 68 (63.3) |  | |  |
| Medicare Wellness | 10 (6.6) | 8 (5.8) | 0.34 | 1 (9.1) | 16 (7.0) | 0.68 | |  |
| Adult Wellness | 49 (32.2) | 34 (24.8) |  | 8 (18.2) | 145 (29.7) |  | |  |
| 1. Percentages based on denominator of all eligible patients of the pilot (N = 289) or column total  2. Percentages based on denominator of all patients who had any response to any questions on the SDOH survey entered into EHR (not including “No response") (N=240) or column total  3. No response to SDOH survey included “Declined” and “Blank”  4. p-values based on Chi-square test statistic or Fisher’s exact test  5. “Other” race/ethnicity category includes American Indian/Alaska Native individuals, Native Hawaiian/Pacific Islander individuals, individuals with multiple racial identities, and individuals whose racial/ethnic identity is unknown or not reported. These groups were combined due to small numbers.  6. “Public” insurance includes both Medicare and Medicaid users  *p<0.05 | | | | | | |  | |

**c. Response to SDOH survey and SDOH identified need across 2020 eligible patient sociodemographic characteristics: Transportation**

|  | **Transportation domain responses^1^** | | | **Transportation domain identified need^2^** | | | |  |
| --- | --- | --- | --- | --- | --- | --- | --- | --- |
|  | **Responded** | **No response^3^** | ***p* values^4^** | **Yes** | **No** | ***p* values^4^** | |  |
|  | N (%) | N(%) |  | N(%) | N(%) |  |  |  |
| **All eligible patients** | 165 (57.1) | 124 (42.9) |  | 6 (2.5) | 234 (97.5) |  | |  |
| **Age** |  |  |  |  |  |  | |  |
| 18 – 24 | 22 (13.3) | 13 (10.5) | 0.59 | 0 (0.0) | 30 (12.8) | 0.19 | |  |
| 25 – 34 | 38 (23.0) | 25 (20.2) |  | 0 (0.0) | 53 (22.6) |  |  |  |
| 35 – 54 | 60 (36.4) | 41 (33.1) |  | 2 (33.3) | 84 (35.9) |  |  |  |
| 55 – 64 | 20 (12.1) | 20 (16.1) |  | 1 (16.7) | 31 (13.2) |  |  |  |
| 65+ | 25 (15.2) | 25 (20.2) |  | 3 (50.0) | 36 (15.4) |  |  |  |
| **Gender** |  |  |  |  |  |  | |  |
| Male | 58 (35.2) | 56 (45.2) | 0.11 | 1 (16.7) | 88 (37.6) | 0.42 | |  |
| Female | 107 (64.9) | 68 (54.8) |  | 5 (83.3) | 146 (62.4) |  |  |  |
| **Race/Ethnicity** |  |  |  |  |  |  | |  |
| Non-Hispanic white | 51 (30.9) | 38 (30.6) | 0.94 | 0 (0.0) | 72 (30.8) | 0.001* | |  |
| Non-Hispanic Black | 35 (21.2) | 22 (17.7) |  | 3 (50.0) | 48 (20.5) |  |  |  |
| Non-Hispanic Asian | 13 (7.9) | 12 (9.7) |  | 3 (50.0) | 17 (7.3) |  |  |  |
| Hispanic/Latinx | 52 (31.5) | 40 (32.3) |  | 0 (0.0) | 72 (30.8) |  |  |  |
| Other^5^ | 14 (8.5) | 12 (9.7) |  | 0 (0.0) | 25 (10.7) |  |  |  |
| **Insurance type** |  |  |  |  |  |  | |  |
| Public^6^ | 33 (20.0) | 25 (20.2) | 1.00 | 3 (50.0) | 43 (18.4) | 0.17 | |  |
| Private | 123 (74.5) | 92 (74.2) |  | 3 (50.0) | 178 (76.0) |  |  |  |
| Self-pay/Not Listed/Other | 9 (5.5) | 7 (5.6) |  | 0 (0.0) | 13 (5.6) |  |  |  |
| **Visit type** |  |  |  |  |  |  | |  |
| New Patient/Transfer | 101 (61.2) | 87 (70.2) | 0.22 | 5 (83.3) | 148 (63.2) | 0.79 | |  |
| Medicare Wellness | 10 (6.1) | 8 (6.5) |  | 0 (0.0) | 17 (7.3) |  |  |  |
| Adult Wellness | 54 (32.7) | 29 (23.4) |  | 1 (16.7) | 69 (29.5) |  |  |  |
| 1. Percentages based on denominator of all eligible patients of the pilot (N = 289) or column total  2. Percentages based on denominator of all patients who had any response to any questions on the SDOH survey entered into EHR (not including “No response") (N=240) or column total  3. No response to SDOH survey included “Declined” and “Blank”  4. p-values based on Chi-square test statistic or Fisher’s exact test  5. “Other” race/ethnicity category includes American Indian/Alaska Native individuals, Native Hawaiian/Pacific Islander individuals, individuals with multiple racial identities, and individuals whose racial/ethnic identity is unknown or not reported. These groups were combined due to small numbers.  6. “Public” insurance includes both Medicare and Medicaid users  *p<0.05 | | | | | | |  | |

**d. Response to SDOH survey and SDOH identified need across 2020 eligible patient sociodemographic characteristics: Alcohol**

|  | **Alcohol domain responses^1^** | | | **Alcohol domain identified need^2^** | | |  |  |
| --- | --- | --- | --- | --- | --- | --- | --- | --- |
|  | **Responded** | **No response^3^** | ***p* values^4^** | **Yes** | **No** | ***p* values^4^** |  |  |
|  | N (%) | N(%) |  | N(%) | N(%) |  |  |  |
| **All eligible patients** | 195 (67.5) | 94 (32.5) |  | 29 (12.1) | 211 (87.9) |  |  |  |
| **Age** |  |  |  |  |  |  |  |  |
| 18 – 24 | 23 (11.8) | 12 (12.8) | 0.048* | 2 (6.9) | 28 (13.3) | 0.73 |  |  |
| 25 – 34 | 45 (23.1) | 18 (19.2) |  | 6 (20.7) | 47 (22.3) |  |  |  |
| 35 – 54 | 75 (38.5) | 26 (27.7) |  | 10 (34.5) | 76 (36.0) |  |  |  |
| 55 – 64 | 27 (13.9) | 13 (13.8) |  | 4 (13.8) | 28 (13.3) |  |  |  |
| 65+ | 25 (12.8) | 25 (26.6) |  | 7 (24.1) | 32 (15.2) |  |  |  |
| **Gender** |  |  |  |  |  |  |  |  |
| Male | 75 (38.5) | 39 (41.5) | 0.72 | 16 (55.2) | 73 (34.6) | 0.05 |  |  |
| Female | 120 (61.5) | 55 (58.5) |  | 13 (44.8) | 138 (65.4) |  |  |  |
| **Race/Ethnicity** |  |  |  |  |  |  |  |  |
| Non-Hispanic white | 57 (29.2) | 32 (29.0) | 0.26 | 14 (48.3) | 58 (27.5) | 0.20 |  |  |
| Non-Hispanic Black | 41 (21.0) | 16 (18.5) |  | 6 (20.7) | 45 (21.3) |  |  |  |
| Non-Hispanic Asian | 17 (8.7) | 8 (8.1) |  | 1 (3.5) | 19 (9.0) |  |  |  |
| Hispanic/Latinx | 58 (29.7) | 34 (29.9) |  | 5 (17.2) | 67 (31.8) |  |  |  |
| Other^5^ | 22 (11.3) | 4 (8.5) |  | 3 (10.3) | 22 (10.4) |  |  |  |
| **Insurance type** |  |  |  |  |  |  |  |  |
| Public^6^ | 33 (16.9) | 25 (26.6) | 0.13 | 7 (24.1) | 39 (18.5) | 0.61 |  |  |
| Private | 152 (78.0) | 63 (67.0) |  | 20 (69.0) | 161 (76.3) |  |  |  |
| Self-pay/Not Listed/Other | 10 (5.1) | 6 (6.4) |  | 2 (6.9) | 11 (5.2) |  |  |  |
| **Visit type** |  |  |  |  |  |  |  |  |
| New Patient/Transfer | 135 (69.2) | 53 (56.4) | 0.06 | 22 (75.9) | 131 (62.1) | 0.42 |  |  |
| Medicare Wellness | 9 (4.6) | 9 (9.6) |  | 1 (3.5) | 16 (7.6) |  |  |  |
| Adult Wellness | 51 (26.2) | 32 (34.0) |  | 6 (20.7) | 64 (30.3) |  |  |  |
| 1. Percentages based on denominator of all eligible patients of the pilot (N = 289) or column total  2. Percentages based on denominator of all patients who had any response to any questions on the SDOH survey entered into EHR (not including “No response") (N=240) or column total  3. No response to SDOH survey included “Declined” and “Blank”  4. p-values based on Chi-square test statistic or Fisher’s exact test  5. “Other” race/ethnicity category includes American Indian/Alaska Native individuals, Native Hawaiian/Pacific Islander individuals, individuals with multiple racial identities, and individuals whose racial/ethnic identity is unknown or not reported. These groups were combined due to small numbers.  6. “Public” insurance includes both Medicare and Medicaid users  *p<0.05 | | | | | | | |  |

**e. Response to SDOH survey and SDOH identified need across 2020 eligible patient sociodemographic characteristics: Physical Activity**

|  | **Physical activity domain responses^1^** | | | **Physical activity domain identified need^2^** | | |  |  |
| --- | --- | --- | --- | --- | --- | --- | --- | --- |
|  | **Responded** | **No response^3^** | ***p* values^4^** | **Yes** | **No** | ***p* values^4^** |  |  |
|  | N (%) | N(%) |  | N(%) | N(%) |  |  |  |
| **All eligible patients** | 168 (58.1) | 121 (41.9) |  | 52 (21.7) | 188 (78.3) |  |  |  |
| **Age** |  |  |  |  |  |  |  |  |
| 18 – 24 | 22 (13.1) | 13 (10.7) | 0.97 | 2 (3.9) | 28 (14.9) | 0.14 |  |  |
| 25 – 34 | 36 (21.4) | 27 (22.3) |  | 10 (19.2) | 43 (22.9) |  |  |  |
| 35 – 54 | 59 (35.1) | 42 (34.7) |  | 19 (36.5) | 67 (35.6) |  |  |  |
| 55 – 64 | 22 (13.1) | 18 (14.9) |  | 10 (19.2) | 22 (11.7) |  |  |  |
| 65+ | 29 (17.3) | 21 (17.4) |  | 11 (21.2) | 28 (14.9) |  |  |  |
| **Gender** |  |  |  |  |  |  |  |  |
| Male | 57 (33.9) | 57 (47.1) | 0.03* | 10 (19.2) | 79 (42.0) | 0.004* |  |  |
| Female | 111 (66.1) | 64 (52.9) |  | 42 (80.8) | 109 (58.0) |  |  |  |
| **Race/Ethnicity** |  |  |  |  |  |  |  |  |
| Non-Hispanic white | 51 (30.4) | 38 (31.4) | 0.63 | 21 (40.4) | 51 (27.1) | 0.26 |  |  |
| Non-Hispanic Black | 37 (22.0) | 20 (16.5) |  | 11 (21.2) | 40 (21.3) |  |  |  |
| Non-Hispanic Asian | 13 (7.7) | 12 (9.9) |  | 4 (7.7) | 16 (8.5) |  |  |  |
| Hispanic/Latinx | 50 (29.8) | 42 (34.7) |  | 14 (26.9) | 58 (30.9) |  |  |  |
| Other^5^ | 17 (10.1) | 9 (7.4) |  | 2 (3.9) | 23 (12.2) |  |  |  |
| **Insurance type** |  |  |  |  |  |  |  |  |
| Public^6^ | 34 (20.2) | 24 (19.8) | 0.99 | 11 (21.2) | 35 (18.6) | 0.50 |  |  |
| Private | 125 (74.4) | 90 (74.4) |  | 40 (76.9) | 141 (75.0) |  |  |  |
| Self-pay/Not Listed/Other | 9 (5.4) | 7 (5.8) |  | 1 (1.9) | 12 (6.4) |  |  |  |
| **Visit type** |  |  |  |  |  |  |  |  |
| New Patient/Transfer | 100 (59.5) | 88 (72.7) | 0.06 | 25 (48.1) | 128 (68.1) | 0.02* |  |  |
| Medicare Wellness | 11 (6.6) | 7 (5.8) |  | 7 (13.5) | 10 (5.3) |  |  |  |
| Adult Wellness | 57 (33.9) | 26 (21.5) |  | 20 (38.5) | 50 (26.6) |  |  |  |
| 1. Percentages based on denominator of all eligible patients of the pilot (N = 289) or column total  2. Percentages based on denominator of all patients who had any response to any questions on the SDOH survey entered into EHR (not including “No response") (N=240) or column total  3. No response to SDOH survey included “Declined” and “Blank”  4. p-values based on Chi-square test statistic or Fisher’s exact test  5. “Other” race/ethnicity category includes American Indian/Alaska Native individuals, Native Hawaiian/Pacific Islander individuals, individuals with multiple racial identities, and individuals whose racial/ethnic identity is unknown or not reported. These groups were combined due to small numbers.  6. “Public” insurance includes both Medicare and Medicaid users  *p<0.05 | | | | | | | |  |

**f. Response to SDOH survey and SDOH identified need across 2020 eligible patient sociodemographic characteristics: Stress**

|  | **Stress domain responses^1^** | | | **Stress domain identified need^2^** | | |  |  |
| --- | --- | --- | --- | --- | --- | --- | --- | --- |
|  | **Responded** | **No response^3^** | ***p* values^4^** | **Yes** | **No** | ***p* values^4^** |  |  |
|  | N (%) | N(%) |  | N(%) | N(%) |  |  |  |
| **All eligible patients** | 163 (56.4) | 126 (43.6) |  | 78 (32.5) | 162 (67.5) |  |  |  |
| **Age** |  |  | 0.67 |  |  | 0.07 |  |  |
| 18 – 24 | 21 (12.9) | 14 (11.1) |  | 15 (19.2) | 15 (9.3) |  |  |  |
| 25 – 34 | 37 (22.7) | 26 (20.6) |  | 15 (19.2) | 38 (23.5) |  |  |  |
| 35 – 54 | 60 (36.8) | 41 (32.6) |  | 32 (41.0) | 54 (33.3) |  |  |  |
| 55 – 64 | 21 (12.9) | 19 (15.1) |  | 7 (9.0) | 25 (15.4) |  |  |  |
| 65+ | 24 (14.7) | 26 (20.6) |  | 9 (11.6) | 30 (18.5) |  |  |  |
| **Gender** |  |  | 0.06 |  |  | 1.00 |  |  |
| Male | 56 (34.4) | 58 (46.0) |  | 29 (37.2) | 60 (37.0) |  |  |  |
| Female | 107 (65.6) | 68 (54.0) |  | 49 (62.8) | 102 (73.0) |  |  |  |
| **Race/Ethnicity** |  |  | 0.86 |  |  | 0.44 |  |  |
| Non-Hispanic white | 48 (29.4) | 41 (32.5) |  | 27 (34.6) | 45 (27.8) |  |  |  |
| Non-Hispanic Black | 35 (21.5) | 22 (17.5) |  | 19 (24.4) | 32 (19.7) |  |  |  |
| Non-Hispanic Asian | 13 (8.0) | 12 (9.5) |  | 5 (6.4) | 15 (9.3) |  |  |  |
| Hispanic/Latinx | 51 (31.3) | 41 (32.5) |  | 22 (28.2) | 50 (30.9) |  |  |  |
| Other^5^ | 16 (9.8) | 10 (8.0) |  | 5 (6.4) | 20 (12.3) |  |  |  |
| **Insurance type** |  |  | 0.74 |  |  | 0.60 |  |  |
| Public^6^ | 31 (19.0) | 27 (21.4) |  | 12 (15.4) | 34 (21.0) |  |  |  |
| Private | 124 (76.1) | 91 (72.2) |  | 62 (79.5) | 119 (73.5) |  |  |  |
| Self-pay/Not Listed/Other | 8 (4.9) | 8 (6.4) |  | 4 (5.1) | 9 (5.5) |  |  |  |
| **Visit type** |  |  | 0.26 |  |  | 0.11 |  |  |
| New Patient/Transfer | 88 (69.8) | 100 (61.3) |  | 55 (70.5) | 98 (60.5) |  |  |  |
| Medicare Wellness | 8 (6.4) | 10 (6.2) |  | 2 (2.6) | 15 (9.3) |  |  |  |
| Adult Wellness | 30 (23.8) | 53 (32.5) |  | 21 (26.9) | 49 (30.2) |  |  |  |
| 1. Percentages based on denominator of all eligible patients of the pilot (N = 289) or column total  2. Percentages based on denominator of all patients who had any response to any questions on the SDOH survey entered into EHR (not including “No response") (N=240) or column total  3. No response to SDOH survey included “Declined” and “Blank”  4. p-values based on Chi-square test statistic or Fisher’s exact test  5. “Other” race/ethnicity category includes American Indian/Alaska Native individuals, Native Hawaiian/Pacific Islander individuals, individuals with multiple racial identities, and individuals whose racial/ethnic identity is unknown or not reported. These groups were combined due to small numbers.  6. “Public” insurance includes both Medicare and Medicaid users  *p<0.05 | | | | | | | |  |

**g. Response to SDOH survey and SDOH identified need across 2020 eligible patient sociodemographic characteristics: Depression**

|  | **Depression domain responses^1^** | | | **Depression domain identified need^2^** | | |  |  |
| --- | --- | --- | --- | --- | --- | --- | --- | --- |
|  | **Responded** | **No response^3^** | ***p* values^4^** | **Yes** | **No** | ***p* values^4^** |  |  |
|  | N (%) | N(%) |  | N(%) | N(%) |  |  |  |
| **All eligible patients** | 20 (6.9) | 269 (93.1) |  | 5 (2.1) | 235 (97.9) |  |  |  |
| **Age** |  |  | < 0.001* |  |  | 0.007* |  |  |
| 18 – 24 | 0 (0) | 35 (13.0) |  | 0 (0) | 30 (12.8) |  |  |  |
| 25 – 34 | 0 (0) | 63 (23.4) |  | 0 (0) | 53 (22.5) |  |  |  |
| 35 – 54 | 0 (0) | 101 (37.5) |  | 0 (0) | 86 (36.6) |  |  |  |
| 55 – 64 | 2 (10.0) | 38 (14.1) |  | 2 (40.0) | 30 (12.8) |  |  |  |
| 65+ | 18 (90.0) | 32 (11.9) |  | 3 (60.0) | 36 (15.3) |  |  |  |
| **Gender** |  |  | 0.51 |  |  | 0.65 |  |  |
| Male | 6 (30.0) | 108 (40.1) |  | 1 (20.0) | 88 (37.4) |  |  |  |
| Female | 14 (70.0) | 161 (59.9) |  | 4 (80.0) | 147 (62.6) |  |  |  |
| **Race/Ethnicity** |  |  | 0.26 |  |  | 1.0 |  |  |
| Non-Hispanic white | 9 (45.0) | 80 (29.7) |  | 2 (40.0) | 70 (29.8) |  |  |  |
| Non-Hispanic Black | 4 (20.0) | 53 (19.7) |  | 1 (20.0) | 50 (21.3) |  |  |  |
| Non-Hispanic Asian | 0 (0) | 25 (9.3) |  | 0 (0) | 20 (8.5) |  |  |  |
| Hispanic/Latinx | 4 (20.0) | 88 (32.7) |  | 2 (40.0) | 70 (29.8) |  |  |  |
| Other^4^ | 3 (15.0) | 23 (8.6) |  | 0 (0) | 25 (10.6) |  |  |  |
| **Insurance type** |  |  | < 0.001* |  |  | < 0.001* |  |  |
| Public^5^ | 20 (100.0) | 38 (14.1) |  | 5 (100.0) | 41 (17.5) |  |  |  |
| Private | 0 (0) | 215 (80.0) |  | 0 (0) | 181 (77.0) |  |  |  |
| Self-pay/Not Listed/Other | 0 (0) | 16 (5.9) |  | 0 (0) | 13 (5.5) |  |  |  |
| **Visit type** |  |  | < 0.001* |  |  | 0.003* |  |  |
| New Patient/Transfer | 0 (0) | 188 (69.9) |  | 0 (0) | 153 (65.1) |  |  |  |
| Medicare Wellness | 15 (75.0) | 3 (1.1) |  | 2 (40.0) | 15 (6.4) |  |  |  |
| Adult Wellness | 5 (25.0) | 78 (29.0) |  | 3 (60.0) | 67 (28.5) |  |  |  |
| 1. Percentages based on denominator of all eligible patients of the pilot (N = 289) or column total  2. Percentages based on denominator of all patients who had any response to any questions on the SDOH survey entered into EHR (not including “No response") (N=240) or column total  3. No response to SDOH survey included “Declined” and “Blank”  4. p-values based on Chi-square test statistic or Fisher’s exact test  5. “Other” race/ethnicity category includes American Indian/Alaska Native individuals, Native Hawaiian/Pacific Islander individuals, individuals with multiple racial identities, and individuals whose racial/ethnic identity is unknown or not reported. These groups were combined due to small numbers.  6. “Public” insurance includes both Medicare and Medicaid users  *p<0.05 | | | | | | | |  |

**h. Response to SDOH survey and SDOH identified need across 2020 eligible patient sociodemographic characteristics: Social connections**

|  | **Social domain responses^1^** | | | **Social domain identified need^2^** | | |  |  |
| --- | --- | --- | --- | --- | --- | --- | --- | --- |
|  | **Responded** | **No response^3^** | ***p* values^4^** | **Yes** | **No** | ***p* values^4^** |  |  |
|  | N (%) | N(%) |  | N(%) | N(%) |  |  |  |
| **All eligible patients** | 159 (55.0) | 130 (45.0) |  | 15 (6.3) | 225 (93.8) |  |  |  |
| **Age** |  |  | 0.59 |  |  | 0.73 |  |  |
| 18 – 24 | 22 (13.8) | 13 (10.0) |  | 1 (6.7) | 29 (12.9) |  |  |  |
| 25 – 34 | 36 (22.6) | 27 (20.8) |  | 5 (33.3) | 48 (21.3) |  |  |  |
| 35 – 54 | 57 (35.8) | 44 (33.8) |  | 6 (40.0) | 80 (35.6) |  |  |  |
| 55 – 64 | 21 (13.2) | 19 (14.6) |  | 2 (13.3) | 30 (13.3) |  |  |  |
| 65+ | 23 (14.5) | 27 (20.8) |  | 1 (6.7) | 38 (16.9) |  |  |  |
| **Gender** |  |  | 0.13 |  |  | 0.42 |  |  |
| Male | 56 (35.2) | 58 (44.6) |  | 7 (46.7) | 82 (36.4) |  |  |  |
| Female | 103 (64.8) | 72 (55.4) |  | 8 (53.3) | 143 (63.6) |  |  |  |
| **Race/Ethnicity** |  |  | 0.80 |  |  | 0.95 |  |  |
| Non-Hispanic white | 48 (30.2) | 41 (31.5) |  | 4 (26.7) | 68 (30.2) |  |  |  |
| Non-Hispanic Black | 35 (22.0) | 22 (16.9) |  | 4 (26.7) | 47 (20.9) |  |  |  |
| Non-Hispanic Asian | 12 (7.6) | 13 (10.0) |  | 1 (6.6) | 19 (8.5) |  |  |  |
| Hispanic/Latinx | 49 (30.8) | 43 (33.1) |  | 4 26.7) | 68 (30.2) |  |  |  |
| Other^5^ | 15 (9.4) | 11 (8.5) |  | 2 (13.3) | 23 (10.2) |  |  |  |
| **Insurance type** |  |  | 0.76 |  |  | 0.10 |  |  |
| Public^6^ | 30 (18.9) | 28 (21.5) |  | 0 (0) | 46 (20.4) |  |  |  |
| Private | 121 (76.1) | 94 (72.3) |  | 15 (100.0) | 166 (73.8) |  |  |  |
| Self-pay/Not Listed/Other | 8 (5.0) | 8 (6.2) |  | 0 (0) | 13 (5.8) |  |  |  |
| **Visit type** |  |  | 0.24 |  |  | 0.69 |  |  |
| New Patient/Transfer | 97 (61.0) | 91 (70.0) |  | 11 (73.3) | 142 (63.1) |  |  |  |
| Medicare Wellness | 10 (6.3) | 8 (6.2) |  | 0 (0) | 17 (7.6) |  |  |  |
| Adult Wellness | 52 (32.7) | 31 (23.8) |  | 4 (26.7) | 66 (29.3) |  |  |  |
| 1. Percentages based on denominator of all eligible patients of the pilot (N = 289) or column total  2. Percentages based on denominator of all patients who had any response to any questions on the SDOH survey entered into EHR (not including “No response") (N=240) or column total  3. No response to SDOH survey included “Declined” and “Blank”  4. p-values based on Chi-square test statistic or Fisher’s exact test  5. “Other” race/ethnicity category includes American Indian/Alaska Native individuals, Native Hawaiian/Pacific Islander individuals, individuals with multiple racial identities, and individuals whose racial/ethnic identity is unknown or not reported. These groups were combined due to small numbers.  6. “Public” insurance includes both Medicare and Medicaid users  *p<0.05 | | | | | | | |  |

**i. Response to SDOH survey and SDOH identified need across 2020 eligible patient sociodemographic characteristics: Intimate partner violence (IPV)**

|  | **IPV domain responses^1^** | | | **IPV domain identified need^2^** | | |  |  |
| --- | --- | --- | --- | --- | --- | --- | --- | --- |
|  | **Responded** | **No response^3^** | ***p* values^4^** | **Yes** | **No** | ***p* values^4^** |  |  |
|  | N (%) | N(%) |  | N(%) | N(%) |  |  |  |
| **All eligible patients** | 162 (56.1) | 127 (43.9) |  | 13 (5.4) | 227 (94.6) |  |  |  |
| **Age** |  |  | 0.66 |  |  | 0.06 |  |  |
| 18 – 24 | 22 (13.6) | 13 (10.2) |  | 5 (38.4) | 25 (11.0) |  |  |  |
| 25 – 34 | 37 (22.8) | 26 (20.4) |  | 3 (23.1) | 50 (22.0) |  |  |  |
| 35 – 54 | 58 (35.8) | 43 (33.9) |  | 2 (15.4) | 84 (37.0) |  |  |  |
| 55 – 64 | 21 (13.0) | 19 (15.0) |  | 2 (15.4) | 30 (13.2) |  |  |  |
| 65+ | 24 (14.8) | 26 (20.5) |  | 1 (7.7) | 38 (16.8) |  |  |  |
| **Gender** |  |  | 0.04* |  |  | 1.0 |  |  |
| Male | 55 (34.0) | 68 (46.5) |  | 5 (38.5) | 84 (37.0) |  |  |  |
| Female | 107 (66.0) | 59 (53.5) |  | 8 (61.5) | 143 (63.0) |  |  |  |
| **Race/Ethnicity** |  |  | 0.97 |  |  | 0.63 |  |  |
| Non-Hispanic white | 49 (30.2) | 40 (31.5) |  | 3 (23.1) | 69 (30.4) |  |  |  |
| Non-Hispanic Black | 34 (21.0) | 23 (18.1) |  | 5 (38.4) | 46 (20.2) |  |  |  |
| Non-Hispanic Asian | 13 (8.0) | 12 (9.4) |  | 0 (0) | 20 (8.8) |  |  |  |
| Hispanic/Latinx | 51 (31.5) | 41 (32.3) |  | 4 (30.8) | 68 (30.0) |  |  |  |
| Other^5^ | 15 (9.3) | 11 (8.7) |  | 1 (7.7) | 24 (10.6) |  |  |  |
| **Insurance type** |  |  | 0.77 |  |  | 1.0 |  |  |
| Public^6^ | 31 (19.2) | 27 (21.3) |  | 2 (15.4) | 44 (19.4) |  |  |  |
| Private | 123 (75.9) | 92 (72.4) |  | 11 (84.6) | 170 (74.9) |  |  |  |
| Self-pay/Not Listed/Other | 8 (4.9) | 8 (6.3) |  | 0 (0) | 13 (5.7) |  |  |  |
| **Visit type** |  |  | 0.23 |  |  | 0.02* |  |  |
| New Patient/Transfer | 99 (61.1) | 89 (70.1) |  | 13 (100.0) | 140 (61.7) |  |  |  |
| Medicare Wellness | 10 (6.2) | 8 (6.3) |  | 0 (0) | 17 (7.5) |  |  |  |
| Adult Wellness | 53 (32.7) | 30 (23.6) |  | 0 (0) | 70 (30.8) |  |  |  |
| 1. Percentages based on denominator of all eligible patients of the pilot (N = 289) or column total  2. Percentages based on denominator of all patients who had any response to any questions on the SDOH survey entered into EHR (not including “No response") (N=240) or column total  3. No response to SDOH survey included “Declined” and “Blank”  4. p-values based on Chi-square test statistic or Fisher’s exact test  5. “Other” race/ethnicity category includes American Indian/Alaska Native individuals, Native Hawaiian/Pacific Islander individuals, individuals with multiple racial identities, and individuals whose racial/ethnic identity is unknown or not reported. These groups were combined due to small numbers.  6. “Public” insurance includes both Medicare and Medicaid users  *p<0.05 | | | | | | | |  |
