# Supplementary material for: Evaluation of a social determinants of health screening questionnaire and workflow pilot within an adult ambulatory clinic
Source: BMC Fam Pract. 2021 Dec 24;22:256. doi: 10.1186/s12875-021-01598-3 (PMC8708511; doi:10.1186/s12875-021-01598-3)
Supplement: Supplementary file 3 — Additional file 3. Length of visit by 2020 eligible patient sociodemographic characteristics. [file 12875_2021_1598_MOESM3_ESM.docx]

**Additional File 3: Length of visit by 2020 eligible patient sociodemographic characteristics**

|  | **Whole visit** | **Checking-in** | **Rooming** | **Exam** |
| --- | --- | --- | --- | --- |
|  | M(SD) | M(SD) | M(SD) | M(SD) |
| **All eligible patients** | 39.75 (13.88) | 7.57 (6.74) | 9.8 (5.16) | 16.22 (8.65) |
| **Age** |  |  |  |  |
| 18 – 24 | 35.23 (12.04) | 6.89 (4.89) | 9.63 (4.79) | 12.21 (6.68) |
| 25 – 34 | 36.32 (12.39) | 6.79 (5.99) | 8.49 (3.79) | 14.66 (6.50) |
| 35 – 54 | 40.78 (12.97) | 7.29 (7.65) | 9.62 (4.74) | 17.31 (8.74) |
| 55 – 64 | 42.00 (16.86) | 8.00 (6.21) | 10.10 (6.50) | 18.97 (10.48) |
| 65+ | 43.36 (14.81) | 9.28 (7.13) | 11.69 (6.09) | 16.59 (9.44) |
| ***p-value*** | 0.0128^1^* | 0.0928^2^ | 0.1633^2^ | 0.0028^2^* |
| **Gender** |  |  |  |  |
| Male | 39.63 (13.33) | 8.23 (7.55) | 9.41 (4.53) | 15.59 (8.41) |
| Female | 39.83 (14.27) | 7.14 (6.14) | 10.05 (5.53) | 16.63 (8.81) |
| ***p-value*** | 0.9060^1^ | 0.1279^2^ | 0.5362^2^ | 0.3210^1^ |
| **Race/Ethnicity** |  |  |  |  |
| Non-Hispanic white | 41.71 (12.85) | 7.29 (5.58) | 10.35 (5.63) | 17.90 (9.26) |
| Non-Hispanic Black | 39.21 (12.97) | 7.89 (6.87) | 9.61 (4.58) | 16.53 (8.08) |
| Non-Hispanic Asian | 40.08 (15.42) | 6.96 (4.65) | 10.25 (5.88) | 15.69 (9.12) |
| Hispanic/Latinx | 38.57 (15.10) | 7.92 (8.35) | 9.36 (4.80) | 14.89 (8.33) |
| Other^3^ | 38.12 (13.50) | 7.19 (5.70) | 9.47 (5.41) | 14.98 (7.99) |
| ***p-value*** | 0.3811^2^ | 0.9956^2^ | 0.7478^2^ | 0.1412^2^ |
| **Insurance type** |  |  |  |  |
| Public^4^ | 43.64 (14.37) | 8.81 (6.73) | 12.20 (6.72) | 17.56 (10.31) |
| Private | 38.52 (13.50) | 7.03 (6.60) | 9.27 (4.49) | 15.80 (8.08) |
| Self-pay/Not Listed/Other | 42.19 (15.11) | 10.44 (7.73) | 8.29 (4.94) | 16.97 (9.61) |
| ***p-value*** | 0.0184^2^* | 0.0125^2^* | 0.0066^2^* | 0.3781^2^ |
| **Visit type** |  |  |  |  |
| New Patient/Transfer | 40.66 (13.92) | 7.78 (7.29) | 10.57 (5.05) | 16.71 (8.62) |
| Medicare Wellness | 43.22 (14.21) | 7.94 (6.58) | 12.7 (6.8) | 15.95 (9.85) |
| Adult Wellness | 36.93 (13.44) | 7.02 (5.39) | 7.43 (4.12) | 15.17 (8.47) |
| ***p-value*** | 0.0525^2^ | 0.8641^2^ | <0.0001^2^* | 0.2278^2^ |
| 1. p-values based on ANOVA  2. p-values based on Kruskal Wallis  3. “Other” race/ethnicity category includes American Indian/Alaska Native individuals, Native Hawaiian/Pacific Islander individuals, individuals with multiple racial identities, and individuals whose racial/ethnic identity is unknown or not reported. These groups were combined due to small numbers.  4. “Public” insurance includes both Medicare and Medicaid users  *p<0.05 | | | | |
